# Primed for death: Law enforcement-citizen homicides, social media, and retaliatory violence

**DOI:** 10.1371/journal.pone.0190571

**Published:** 2018-01-10

**Authors:** Vladimir Bejan, Matthew Hickman, William S. Parkin, Veronica F. Pozo

**Affiliations:** 1 Department of Economics, Seattle University, Seattle, WA 98122, United States of America; 2 Department of Criminal Justice, Seattle University, Seattle, WA 98122, United States of America; 3 Department of Applied Economics, Utah State University, Logan, UT 84322, United States of America; University of Pennsylvania, UNITED STATES

## Abstract

We examine whether retaliatory violence exists between law enforcement and citizens while controlling for any social media contagion effect related to prior fatal encounters. Analyzed using a trivariate dynamic structural vector-autoregressive model, daily time-series data over a 21-month period captured the frequencies of police killed in the line of duty, police deadly use of force incidents, and social media coverage. The results support a significant retaliatory violence effect against minorities by police, yet there is no evidence of retaliatory violence against law enforcement officers by minorities. Also, social media coverage of the Black Lives Matter movement increases the risk of fatal victimization to both law enforcement officers and minorities. Possible explanations for these results are based in rational choice and terror management theories.

## Introduction

Fatal interactions between police and the public became the subject of increased scrutiny following the 2014 shooting of Michael Brown in Ferguson, Missouri, and several subsequent controversial police shootings. These fatal interactions are objectively rare in the context of all contacts between police and the communities they serve, but they can evoke strong emotional reactions from the public. They are typically complex and dynamic events, the details are slow to emerge, and to the public the legal structure and process surrounding these events are confusing, if not frustrating. Police use of deadly force is perhaps the most powerful expression of state authority and it can be equally powerful in undermining the public perception of police legitimacy.

The Black Lives Matter (BLM) movement, which emerged after the shooting of Trayvon Martin by Florida resident George Zimmerman, has seen an increased profile since the shooting of Michael Brown and related public demonstrations and riots. The developing narrative reflects a belief that police officers are more likely to kill minorities, all else being equal, and that the law insulates law enforcement from accountability. As a movement there is little evidence that BLM supporters endorse retaliatory violence against the police. However, several police leaders have publicly expressed their opinion that BLM clearly does endorse such violence. This narrative includes claims that the police are under attack and that there is a “war on cops.” The killing of five Dallas police officers in 2016 is a particularly troubling touch point in this regard. While there has been debate over the nature of the threat, it can be said that the number of shootings of police during 2016 has exceeded the number recorded in 2015 [1].

Conservative political commentator Heather MacDonald is often credited with articulating the “Ferguson Effect” hypothesis, asserting that police officers respond to their fears of increased scrutiny by engaging in less proactive policing, leading to increased crime [2]. The hypothesis has been extended to encompass a more general hesitancy to act, leading to decreased officer safety, as well as increases in retaliatory lethal attacks on police to avenge what are seen as racially-motivated killings of blacks. One anecdotal example of this phenomenon was the stated refusal of a Chicago police officer to draw her weapon while being beaten because she feared the media coverage if she shot and killed her assailant, who was an African-American male [3].

This situation has led several scholars to investigate whether there were changes in proactive policing reflected in increased crime [4], and debate whether violence by and/or toward the police has increased, decreased, or stayed the same in the wake of these events. Recent research has examined whether there was a detectable increase in killings of police post-Ferguson (through March, 2016) and found no evidence to support the hypothesis [5]. While it is important to assess the effects of Ferguson and BLM as a historical turning point in perceptions of police legitimacy, as well as subsequent police and public behavior, the broader and arguably more important question that this research addresses is the general process question: whether the taking of life by law enforcement is associated with retaliatory lethal violence against law enforcement and whether the reverse may also be true, that lethal violence against law enforcement is associated with increased killings by police. In fact, signals of perceived culpability for this violence and who is responsible can be found in other areas of our criminal justice system. In the courts, representatives of the law enforcement community have sued the Black Lives Matter movement and other organizations, claiming they are responsible for violence against officers. In one instance, the father of a Dallas Police Officer killed in an ambush by an offender specifically targeting law enforcement, sued the Black Lives Matter movement and others, who the complainant argues “have convinced their supporters that there is a civil war between blacks and law enforcement, thereby calling for immediate violence and severe bodily injury or death” [6]. A similar suit “claims Black Lives Matter leaders incited others to harm police in retaliation for the death of black men killed by police” [7].

The United States has a long history and tradition of local control over law enforcement; this has manifested in nearly 18,000 state and local law enforcement agencies that operate outside of any unified command and control structure. As such, the best way to model public and law enforcement knowledge of lethal interactions—a prerequisite to retaliatory violence—is through media coverage. Over the last two years the media in general, and social media in particular, have been increasingly used to inform the public of violence between law enforcement and the public. In a more recent editorial, Heather MacDonald quotes New York Police Commissioner James O’Neill as stating that a suspect who killed a police officer “‘hated the police’…because he had heard and read ‘countless times’ in conversation, on television and in the newspapers that cops were the ‘bad guys.”’ [8]. This reference to the perceived power of the media, and given the rise of social media and its centrality to organizing movements such as BLM, demonstrates why it is important to assess the role of media, if any, in these fatal interactions. This research examines these relationships, and seeks to answer the questions of whether there is any evidence of retaliatory violence between the two groups of victims, and to what extent social media plays a role in inciting these acts of violence.

## Fatal law enforcement & civilian encounters

Research focused on police shootings and criminal homicide is limited. A literature search yielded about a dozen published studies that have empirically assessed this relationship since 1970. A significant problem that persists to the present day is the lack of adequate data about police shootings. While the government has invested in collecting detailed information about officers killed in the line of duty, there has been less investment in collecting data on police use of force and justifiable and non-justifiable homicides by police. This has led to recent data collection efforts by media organizations such as *The Guardian* and *The Washington Post*, as well as crowd-sourced efforts such as www.killedbypolice.net, to enumerate individuals killed by law enforcement. The utility of these data collection efforts is demonstrated by the Justice Department’s recent reliance upon them to improve the validity and reliability of their own data [9].

The earliest reported U.S. studies relied on either Vital Statistics data or administrative data for either a single agency or a small collection of agencies, and typically used cross-sectional designs and bivariate analytic methods (see [10–15]). These early studies were fairly consistent in finding a relationship between police shootings and criminal homicide, but were unable to draw conclusions about the direction of the relationship. The first reported longitudinal analysis, conducted in 1986 using NYPD data, concluded that the bivariate relationships reported in the literature were likely spurious [16].

In the early 1990’s, researchers turned their attention toward the FBI’s Supplemental Homicide Reports (SHR) and subsequent research has consistently reported finding a relationship between various measures of community violence and police shootings (see [17–20]). One longitudinal study, conducted in 2001 using SHR data, reported a temporal relationship between predatory homicides and police shootings at the national level [21]. However, it is important to note that the time-series analysis used to model the relationship implicitly assumes that there is no feedback between variables. Specifically, it models police shootings as a function of predatory homicides, ignoring the fact that police homicides can, in turn, impact levels of predatory homicide. This is an inherent problem with this type of time-series analysis and in most instances represents a misspecification of the model [22].

In sum, the current state of knowledge about the temporal relationship between police shootings and criminal homicide is extremely limited. While there is evidence of a bivariate relationship between violence and police shootings across time, location, levels of analysis, data sources, analytic methods, and theoretical frameworks, absent from the literature is a longitudinal study that accounts for the cyclical relationship between the variables under study as well as competing explanatory variables, such as media coverage as contagion.

## Social media as contagion

There is evidence that the media can act as a contagion, not only spreading ideas and information, but also emotions. For this study, we reason that for there to be retaliatory violence, citizens and law enforcement must be aware of the initial acts of violence. If a minority citizen is shot and killed by police and there is no media coverage of the event, it is difficult to argue that the killing of a law enforcement officer a day or a week later is driven by the prior fatal encounter. It is therefore important to understand how social media coverage of such events might communicate information about deadly encounters across social networks.

Both traditional and social media can act as an emotional contagion, spreading fear, anger and other negative affects in addition to pure information. Social media has been shown to directly impact the emotions of those consuming it. For example, looking at the social media activity of millions of Facebook users, it has been demonstrated that rain decreased the frequency of posts with a positive connotation while increasing those with a negative connotation [23]. Importantly, the researchers also found that an increase in user’s negative posts significantly decreased the frequency of positive posts by individuals within their social networks, even though those friends were not physically located in areas with inclement weather. An experimental study that manipulated the Facebook content provided to hundreds of thousands of users found that reducing the number of positive posts to which users were exposed significantly decreased the frequency of positive words they used in subsequent posts, and a similar effect was found for decreasing posts with a negative connotation leading to the use of fewer negative words [24]. These studies provide empirical support that a person’s emotional state and, subsequently, how they express themselves can be directly impacted by their social media networks.

In addition, research has demonstrated that tweets with a negative or positive sentiment are retweeted more often and faster than tweets with a neutral message [25]. This behavior could impact the breadth and speed of dissemination of social media messages related to fatal encounters between law enforcement and the public. Due to the emotionally charged nature of BLM for supporters and non-supporters alike, one would expect that tweets referencing the movement would be anything but neutral in their content. This has implications for the contagion of social media and could explain why #BlackLivesMatter was one of the top hashtags used in 2015, a period that encompasses almost two-thirds of the time period under study for this research [26].

This evidence of social media contagion and the spread of negative emotion is important when viewed through the lens of General Strain Theory (GST) [27–30]. From this perspective, fatal law enforcement-citizen encounters perceived as illegitimate are a source of strain (negative stimuli) generating negative emotions (primarily anger). Due to the particularly unjust nature and high magnitude of this strain (as spread through social media), it is likely that these negative emotions will catalyze negative behaviors (such as retaliatory aggression) in order to reduce the strain. Individual traits and group pressures may also amplify the likelihood that this strain leads to retaliatory aggression. While tests of GST are somewhat mixed, there is ample support for these mechanisms.

Although research on media effects has not specifically examined the relationship between social media and fatal law enforcement-citizen encounters, it does provide evidence that social media coverage of these events could potentially increase the likelihood of future events. To further our understanding of the phenomenon, we explore whether there is evidence that violence between law enforcement and the public is retaliatory in nature and whether there is a media contagion effect that heightens the risk of further violence.

## Research design & measures

We use a structural vector-autoregressive (SVAR) model to analyze the contemporaneous, cyclical and dynamic relationships between variables. The SVAR model controls for temporal ordering and accounts for the relationship that these variables have on each other during the same day (i.e., without a lag). For additional background and technical detail on SVAR models, please see the Supporting information section. Although the SVAR model is considered by some to facilitate drawing causal inferences from time series data, we caution readers that the strongest causal arguments are drawn from experimental methodologies and therefore our results should be interpreted as associations between variables, not causal relationships. For our analysis, we examined four variables across two models.

- LAW: The number of law enforcement officers shot to death per day
- MINORITIES: The number of minorities shot to death by law enforcement per day
- WHITES: The number of white non-Hispanics shot to death by law enforcement per day
- TWITTER: The number of tweets with #BlackLivesMatter or “Black Lives Matter” that were posted per day

The number of law enforcement officers shot in the line of duty was taken from data provided by the *Officer Down Memorial Page* [31], which is made publicly available through their website under fair use for educational purposes. This database allows us to identify the number of officers who were killed by non-accidental gunfire from January 1, 2015 through September 30, 2016, the time period under study for this research. We focus specifically on the number of officers shot to death to mirror the data we used for citizens killed, which also focuses specifically on interactions where individuals are shot during fatal law enforcement-citizen encounters.

The number of individuals killed by law enforcement was taken from *The Washington Post* database tracking citizens shot and killed during interactions with law enforcement in the United States [32], which is made publicly available on their website under a creative commons license. The data are collected through news reports, Internet reports, and public records. In addition to identifying those killed, *The Washington Post* also collects data on victim demographics, most important to this research being race and ethnicity. This variable was used to divide the list of victims into two categories—minorities shot and killed by law enforcement and white non-Hispanics shot and killed by law enforcement. If the race or ethnicity was unknown, these individuals were counted as white non-Hispanic, as the lack of an identifiable minority status would in theory be treated by the BLM movement similarly to the shooting death of a white non-Hispanic. The number of minority victims and white non-Hispanic victims were then aggregated to a daily count for the analysis. For both datasets, it is important to acknowledge the limitations. Each dataset collects data solely on individuals who are shot and killed, which will not represent non-shooting deaths that also may have had a high profile. For example, the deaths of Freddie Gray and Sandra Bland who died while in custody of state actors in the criminal justice system. These deaths were acknowledged by the Black Lives Matter movement, but yet are not represented in our data.

To measure social media exposure, we used Twitter data as a proxy for immediate, social media reaction to law enforcement/citizen violence. Twitter was chosen over other social media services, such as Facebook, as all tweets are accessible to the public, regardless of whether someone is a “friend” or “follows” a user. We used Google advanced search to identify tweets that included either the the entire phrase “Black Lives Matter” or the hashtag #BlackLivesMatter. The advanced search allowed us to focus only on results that were connected to www.twitter.com, had the word “status” in the URL (limiting the results to posts), and occurred on a specific day using Twitter’s date abbreviation format (e.g. “1 Jan 2015” or “30 Sep 2016”). The final search string resembled the following:

(“1 Jan 2015”) AND (“blacklivesmatter” | “black lives matter”) AND (site:twitter.com) AND (inurl:status)

Each search resulted in a count (provided by Google) of results returned based on the advanced search string. These tweets could support, oppose, or be neutral of the Black Lives Matter movement. They are best thought of as a proxy for the amount of social media attention focused on law enforcement and citizen encounters that end in a death, especially where the citizen was a racial or ethnic minority. The results were disaggregated into a single count for each day under study. However, as one reviewer pointed out, the data we use weighs all tweets the same, regardless of the number of consumers for each tweet. For example, an individual with five followers who tweeted using #BlackLivesMatter has the same effect on the model as a twitter handle who tweeted using the same hashtag that had five million followers. Similarly, our measure of social media does not control for the frequency of tweets by each twitter account and therefore prolific tweeters will have a larger impact on the model, regardless of their reach. Finally, our data does not measure whether the tweets are actually about the death of a citizen through lethal use of force or the death of an officer in the line of duty. These are inherent limitations to the data we collected.

Table 1 shows the summary statistics for the variables used in the analysis. One can observe that the data is dominated by zeros. To deal with this issue we follow the approach proposed by Burbrdge, Magee and Robb (1988) [33], who expand on Johnson (1949) [34], and perform an inverse hyperbolic sine (IHS) transformation on each of the homicide count variables, as follows:

$$\begin{array}{c} g_{t}=\text{ln}\left(\theta Y_{t}+\sqrt{1+\theta^{2}Y_{t}^{2}}\right) \end{array}$$ (1)

where *g*_*t*_ is the transformed variable and *θ* is set equal to one. The IHS transformation is used “… to reduce the influence of extreme observations of the dependent variable on the regression” [33]. Therefore, we can run linear regression models on the transformed data and interpret the coefficients as if we were using a natural log transformation (i.e., in percentages). This transformation was applied to all data series, including Twitter data, to maintain consistency.

**Table 1:** Model variables summary statistics.

	*Law*	*Minorities*	*Whites*	*Twitter*
min	0	0	0	937
1Q	0	0	1	1720
median	0	1	1	3320
mean	0.1268	1.238	1.449	4991
3Q	0	2	2	5125
max	5	6	6	56000
sd	0.3974	1.1650	1.9960	5606.18

Table 2 shows the summary statistics for the transformed variables. The following plots show that the IHS transformation of homicide rate preserves the properties of the original data.

**Table 2:** Model variables summary statistics.

	*Law*	*Minorities*	*Whites*	*Twitter*
min	0	0	0	7.536
1Q	0	0	0.2177	8.143
median	0	0.8814	0.5623	8.801
mean	0.1059	0.8738	0.6725	8.849
3Q	0	1.1436	0.9371	9.235
max	2.3125	2.4918	2.3120	11.630
sd	0.3063	0.6750	0.5748	0.7913

**Fig 1:**
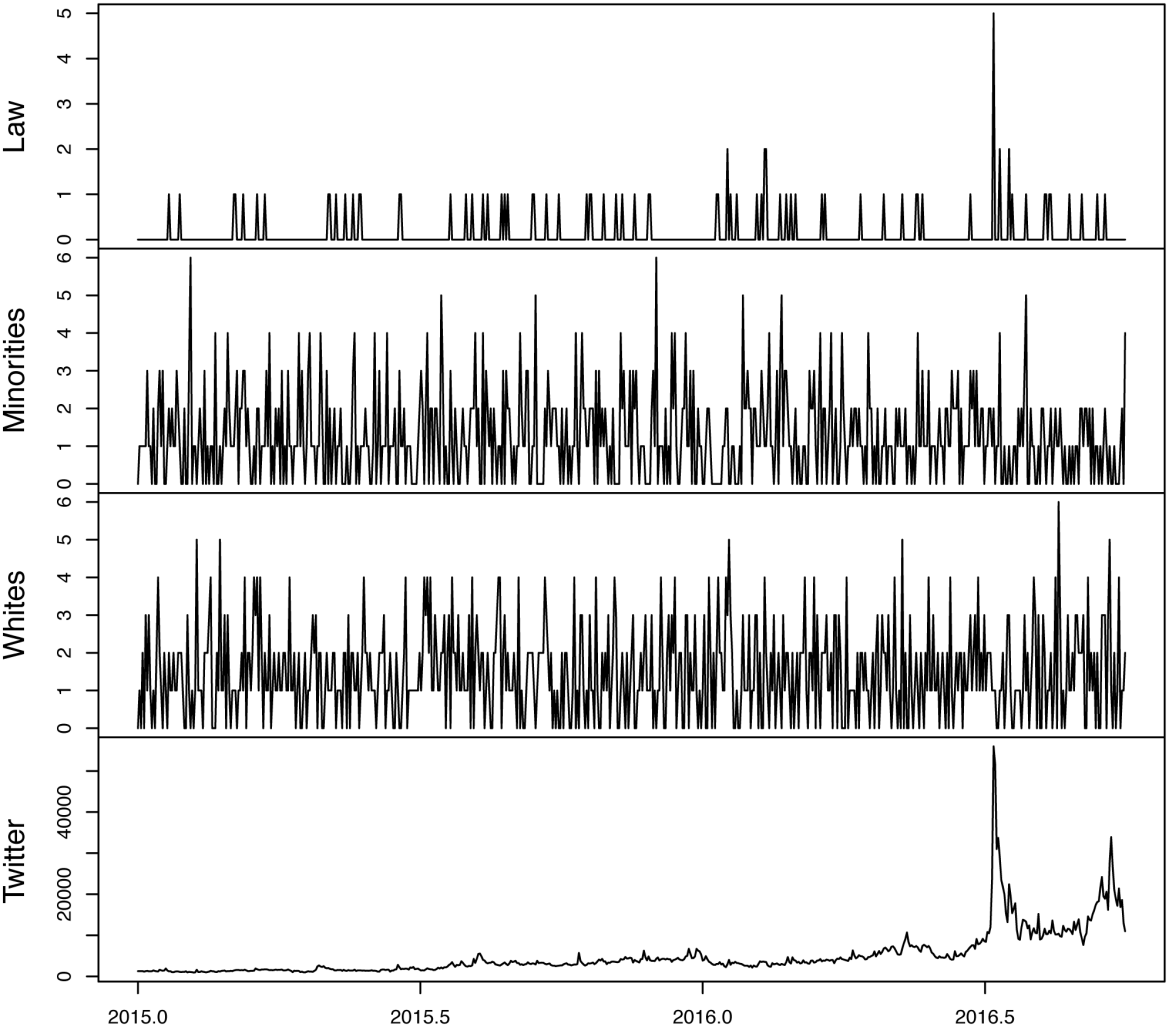
Raw data.

**Fig 2:**
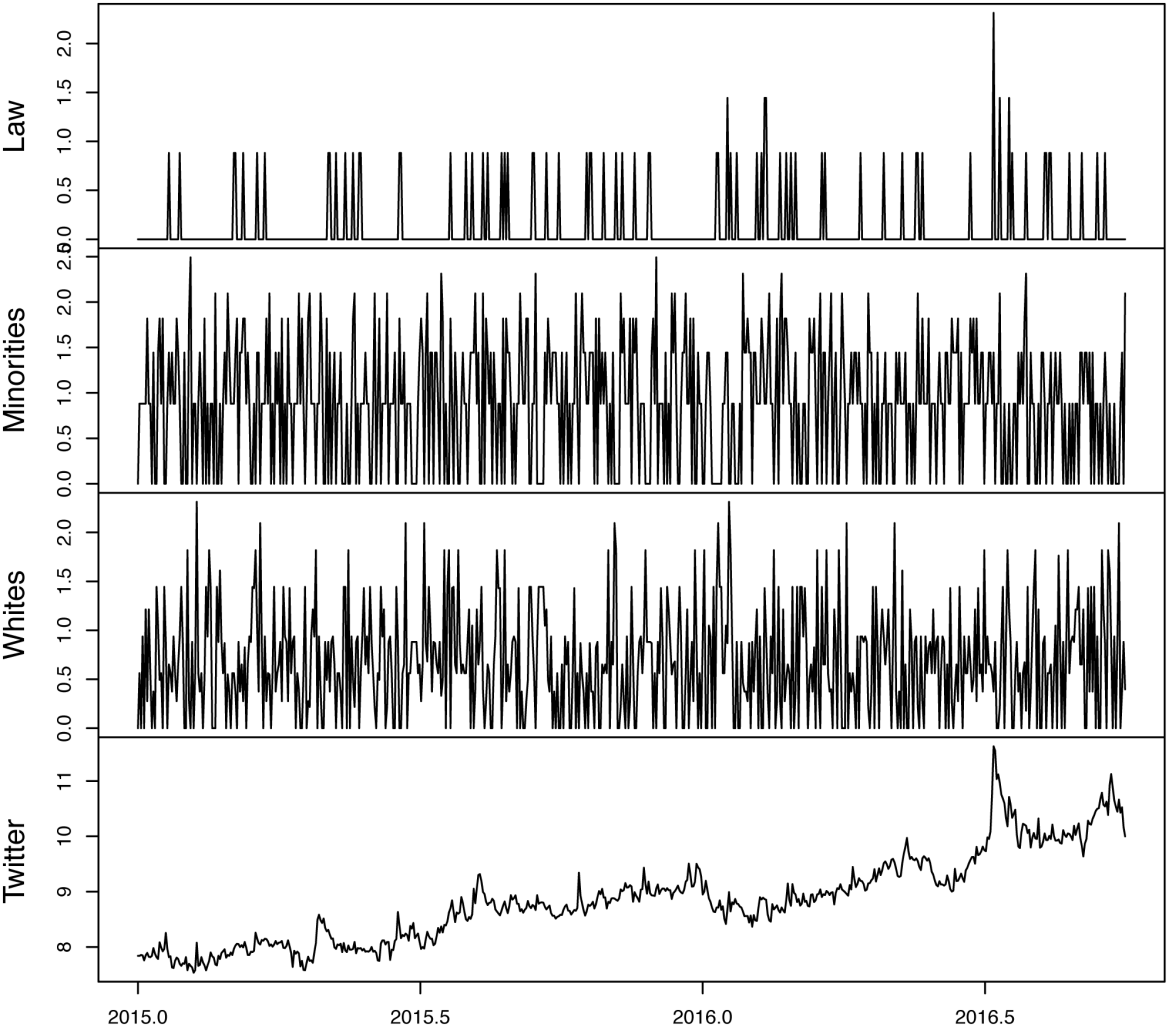
IHS transfromed data.

**Fig 3:**
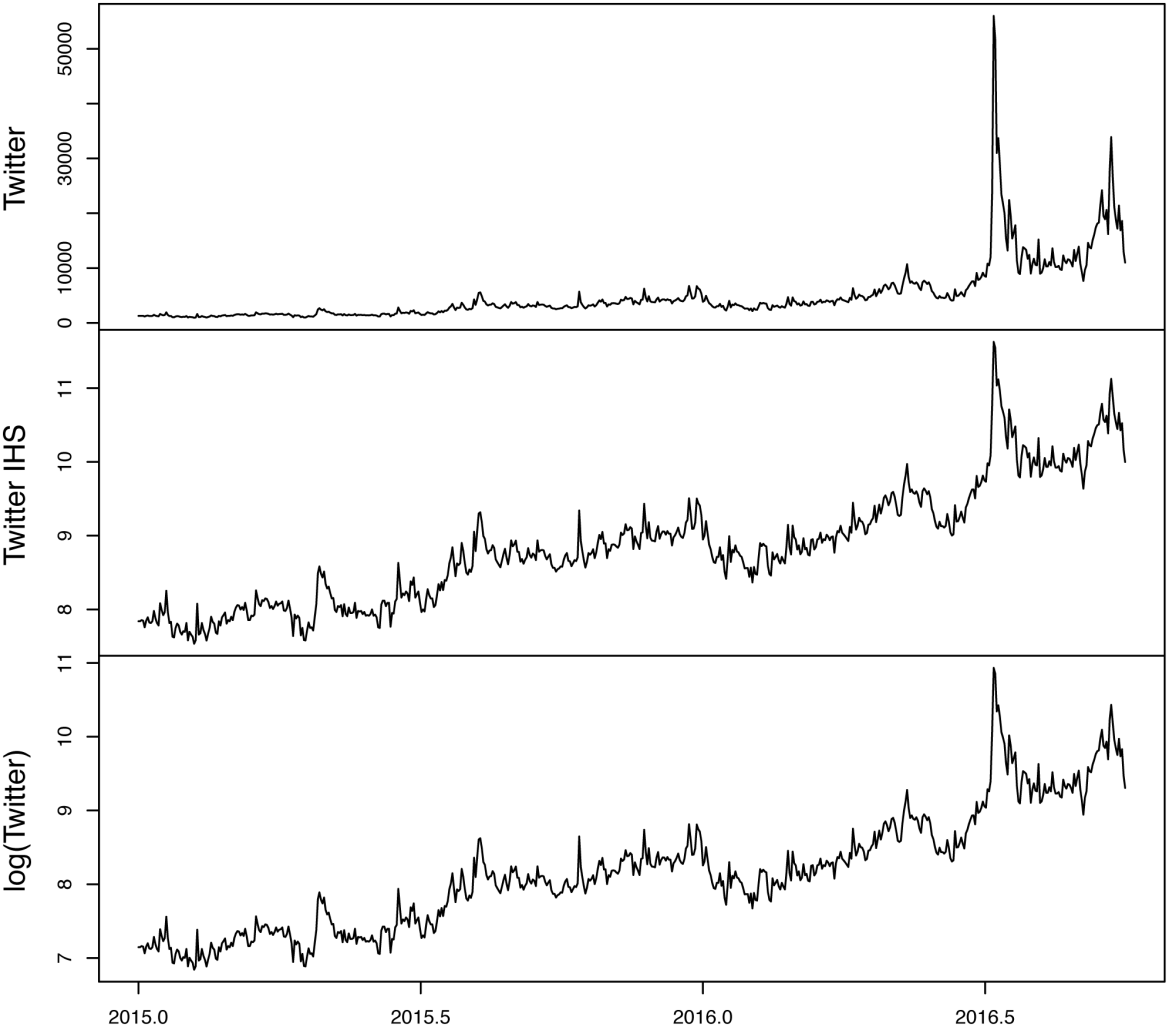
Tweeter data: Raw, IHS transformed and natural log.

Figs 1 and 2 plot the original and transformed series, respectively. Comparing the plots at the bottom of each figure there are scaling differences between the original Twitter data and the IHS transformed data. This is because the latter is equivalent to the natural log transformation of the earlier. To see the equivalency more clearly, Fig 3 plots the raw twitter data, the IHS transformed data and the natural log of the raw twitter data. Notice the similarity between the IHS transformed data and the natural log of the original data.

### Stationarity tests

To appropriately estimate time series models, particularly the one proposed in this research paper, it is imperative to work with stationary series. To test whether each time series is stationarity, we used an Augmented Dickey-Fuller (ADF) test and a KPSS test [35]. The null hypothesis under the ADF test is non-stationary, and we look for evidence to reject such a claim. It is a well known fact that the power of such test is relatively low because it is biased toward not rejecting the null even if the data generating process is stationary. Also, the power of this test diminishes even more when deterministic components such as intercept and trend are added to the model [36]. Therefore, we use the KPSS test as a robustness check. On the contrary, the null hypothesis under the KPSS test is stationarity. Table 3 shows the results from both tests. All series are stationary except for Twitter data. Because the Twitter series is non-stationary, it needs to be transformed (i.e., differenced).

**Table 3:** Unit root tests.

	ADF (drift)				KPSS			
	*test*.*stat*	95% *crit*.*val*	*lags*^(a)^	*stationary*	*test*.*stat*	95% *crit*.*val*	*lags*^(b)^	*stationary*
*Law*	-16.49	-2.86	1	yes	0.2214	0.463	6	yes
*Minorities*	-15.96	-2.86	1	yes	0.0794	0.463	6	yes
*Whites*	-18.19	-2.86	1	yes	0.0406	0.463	6	yes
*Twitter*	-1.92	-2.86	1	no	7.67	0.463	6	no

(*a*) Lags selected using BIC.

(*b*) Number of lags set used $\sqrt[{4}]{4\times (n/100)}$. See [37].

## Econometric model estimation and results

To examine the relationship between law enforcement killed by citizens, citizens killed by police (minorities and whites) and “Black Lives Matter” (BLM) twits, we start with the following reduced-form model:

$$\begin{array}{c} \begin{array}{c} {\text{law}}_{t}=\alpha_{10}+\sum_{k=1}^{p}\alpha_{2k}{\text{law}}_{t-k}+\sum_{k=1}^{p}\alpha_{3k}{\text{ctz}}_{t-k}+\sum_{k=1}^{p}\alpha_{4k}{\text{twt}}_{t-k}+e_{\text{law},t}\\ {\text{ctz}}_{t}=\beta_{10}+\sum_{k=1}^{p}\beta_{2k}{\text{law}}_{t-k}+\sum_{k=1}^{p}\beta_{3k}{\text{ctz}}_{t-k}+\sum_{k=1}^{p}\beta_{4k}{\text{twt}}_{t-k}+e_{\text{ctz},t}\\ {\text{twt}}_{t}=\gamma_{10}+\sum_{k=1}^{p}\gamma_{2k}{\text{law}}_{t-k}+\sum_{k=1}^{p}\gamma_{3k}{\text{ctz}}_{t-k}+\sum_{k=1}^{p}\gamma_{4k}{\text{twt}}_{t-k}+e_{\text{twt},t} \end{array} \end{array}$$ (2)

where *law* is the number of law enforcement killed by citizens per day, *ctz* = (*minorities*, *whites*) is the number of citizens killed by law enforcement per day, *twt* is the first difference of the number of “Black Lives Matter” tweets per day, and *e*_law,*t*_, *e*_ctz,*t*_ and *e*_twt,*t*_ are the reduced-form residuals. In matrix form, system (2) can be re-written as:

$$\begin{array}{c} Z_{t}=D_{0}+D_{1}Z_{t-1}+\ldots +D_{p}Z_{t-p}+e_{t} \end{array}$$ (3)

where $Z_{t}=\left[\begin{array}{c} {\text{law}}_{t}\\ {\text{ctz}}_{t}\\ {\text{twt}}_{t} \end{array}\right]$, $D_{0}=\left[\begin{array}{c} \alpha_{10}\\ \beta_{10}\\ \delta_{10} \end{array}\right]$, $D_{k}=\left[\begin{array}{ccc} \alpha_{2k} & \alpha_{3k} & \alpha_{4,k}\\ \beta_{2k} & \beta_{3k} & \beta_{4k}\\ \gamma_{2k} & \gamma_{3k} & \gamma_{4k} \end{array}\right]\;\forall \;k=1,\ldots ,p$, $e_{t}=\left[\begin{array}{c} e_{\text{law},t}\\ e_{\text{ctz},t}\\ e_{\text{twt},t} \end{array}\right]$ and *p* is the lag order selected based on the Akaike Information Criterion (AIC). The problem of working with a reduced-form model is that the effect of a one-time shock to any of the variables in the system does not convey any information about the dynamics of the variables under study because reduced-form shocks are correlated. That is, they are composites of the structural shocks. The structural shocks, on the other hand, are orthogonal and therefore uncorrelated by design. This relationship is represented as *e*_*t*_ = *A*^−1^*ε*_*t*_. In matrix form:

$$\begin{array}{c} \left[\begin{array}{c} e_{\text{law},t}\\ e_{\text{ctz},t}\\ e_{\text{twt},t} \end{array}\right]=A^{-1}\left[\begin{array}{c} \varepsilon_{\text{law},t}\\ \varepsilon_{\text{ctz},t}\\ \varepsilon_{\text{twt},t} \end{array}\right] \end{array}$$ (4)

where *ε*_law,*t*_, *ε*_ctz,*t*_ and *ε*_twt,*t*_ are the corresponding structural shocks, and $A^{-1}=\left[\begin{array}{ccc} 1 & \delta_{12} & \delta_{13}\\ \delta_{21} & 1 & \delta_{23}\\ \delta_{31} & \delta_{32} & 1 \end{array}\right]$ is the matrix of contemporaneous effects.

To disentangle the effects of structural shocks, which are uncorrelated, it is necessary to recover the elements of *A*^−1^ (this is commonly referred to as the identification problem). The identification approach used in this study, which was first introduced by Rigobon [38] and is based on the heteroskedasticity of structural shocks, allows as to achieve this goal by identifying regimes of high and low volatility in second moments. Under the assumption of homoskedasticity, the variance-covariance matrix derived from Eq (4) can be specified as follows:

$$\begin{array}{c} \left\{ \begin{array}{c} \text{var}\left(e_{\text{law}}\right)=\text{var}\left(\varepsilon_{\text{law}}\right)+\delta_{12}^{2}\text{var}\left(\varepsilon_{\text{ctz}}\right)+\delta_{13}^{2}\text{var}\left(\varepsilon_{\text{twt}}\right)\\ \text{cov}\left(e_{\text{law}},e_{\text{ctz}}\right)=\delta_{21}\text{var}\left(\varepsilon_{\text{law}}\right)+\delta_{12}\text{var}\left(\varepsilon_{\text{ctz}}\right)+\delta_{13}\delta_{23}\text{var}\left(\varepsilon_{\text{twt}}\right)\\ \text{cov}\left(e_{\text{law}},e_{\text{twt}}\right)=\delta_{31}\text{var}\left(\varepsilon_{\text{law}}\right)+\delta_{12}\delta_{32}\text{var}\left(\varepsilon_{\text{ctz}}\right)+\delta_{13}\text{var}\left(\varepsilon_{\text{twt}}\right)\\ \text{var}\left(e_{\text{ctz}}\right)=\delta_{21}^{2}\text{var}\left(\varepsilon_{\text{law}}\right)+\text{var}\left(\varepsilon_{\text{ctz}}\right)+\delta_{23}^{2}\text{var}\left(\varepsilon_{\text{twt}}\right)\\ \text{cov}\left(e_{\text{ctz}},e_{\text{twt}}\right)=\delta_{21}\delta_{31}\text{var}\left(\varepsilon_{\text{law}}\right)+\delta_{32}\text{var}\left(\varepsilon_{\text{ctz}}\right)+\text{var}\left(\varepsilon_{\text{twt}}\right)\\ \text{var}\left(e_{\text{twt}}\right)=\delta_{31}^{2}\text{var}\left(\varepsilon_{\text{law}}\right)+\delta_{32}^{2}\text{var}\left(\varepsilon_{\text{ctz}}\right)+\text{var}\left(\varepsilon_{\text{twt}}\right) \end{array}\right. \end{array}$$ (5)

Notice that system (5) has 6 equations and 9 unknowns: *ε*_law_, *ε*_ctz_, *ε*_twt_, *δ*_12_, *δ*_13_, *δ*_21_, *δ*_23_, *δ*_31_, and *δ*_32_. Therefore, it cannot be consistently estimated. By identifying more than one regime in the variances of structural shocks, it is possible to solve system (5) without additional restrictions. That is, we can estimate the following system, where *i* = 1, 2 denotes the regime:

$$\begin{array}{c} \left\{ \begin{array}{c} \text{var}\left(e_{\text{law}}^{\left(i\right)}\right)=\text{var}\left(\varepsilon_{\text{law}}^{\left(i\right)}\right)+\delta_{12}^{2}\text{var}\left(\varepsilon_{\text{ctz}}^{\left(i\right)}\right)+\delta_{13}^{2}\text{var}\left(\varepsilon_{\text{twt}}^{\left(i\right)}\right)\\ \text{cov}\left(e_{\text{law}}^{\left(i\right)},e_{\text{ctz}}^{\left(i\right)}\right)=\delta_{21}\text{var}\left(\varepsilon_{\text{law}}^{\left(i\right)}\right)+\delta_{1,2}\text{var}\left(\varepsilon_{\text{ctz}}^{\left(i\right)}\right)+\delta_{13}\delta_{23}\text{var}\left(\varepsilon_{\text{twt}}^{\left(i\right)}\right)\\ \text{cov}\left(e_{\text{law}}^{\left(i\right)},e_{\text{twt}}^{\left(i\right)}\right)=\delta_{31}\text{var}\left(\varepsilon_{\text{law}}^{\left(i\right)}\right)+\delta_{12}\delta_{32}\text{var}\left(\varepsilon_{\text{ctz}}^{\left(i\right)}\right)+\delta_{13}\text{var}\left(\varepsilon_{\text{twt}}^{\left(i\right)}\right)\\ \text{var}\left(e_{\text{ctz}}^{\left(i\right)}\right)=\delta_{21}^{2}\text{var}\left(\varepsilon_{\text{law}}^{\left(i\right)}\right)+\text{var}\left(\varepsilon_{\text{ctz}}^{\left(i\right)}\right)+\delta_{23}^{2}\text{var}\left(\varepsilon_{\text{twt}}^{\left(i\right)}\right)\\ \text{cov}\left(e_{\text{ctz}}^{\left(i\right)},e_{\text{twt}}^{\left(i\right)}\right)=\delta_{21}\delta_{31}\text{var}\left(\varepsilon_{\text{law}}^{\left(i\right)}\right)+\delta_{32}\text{var}\left(\varepsilon_{\text{ctz}}^{\left(i\right)}\right)+\text{var}\left(\varepsilon_{\text{twt}}^{\left(i\right)}\right)\\ \text{var}\left(e_{\text{twt}}^{\left(i\right)}\right)=\delta_{31}^{2}\text{var}\left(\varepsilon_{\text{law}}^{\left(i\right)}\right)+\delta_{32}^{2}\text{var}\left(\varepsilon_{\text{ctz}}^{\left(i\right)}\right)+\text{var}\left(\varepsilon_{\text{twt}}^{\left(i\right)}\right) \end{array}\right. \end{array}$$ (6)

In this case, system (6) has 12 equations and 12 unknowns, which allows us to solve for the structural parameters (contemporaneous coefficients and variances). This method is similar to using instrumental variables to solve for the endogeneity problem. The difference is that it is applied to the variance-covariance matrix of reduced-form residuals [38]. Here, an instrument is the additional heteroskedasticity regime that has been identified.

**Fig 4:**
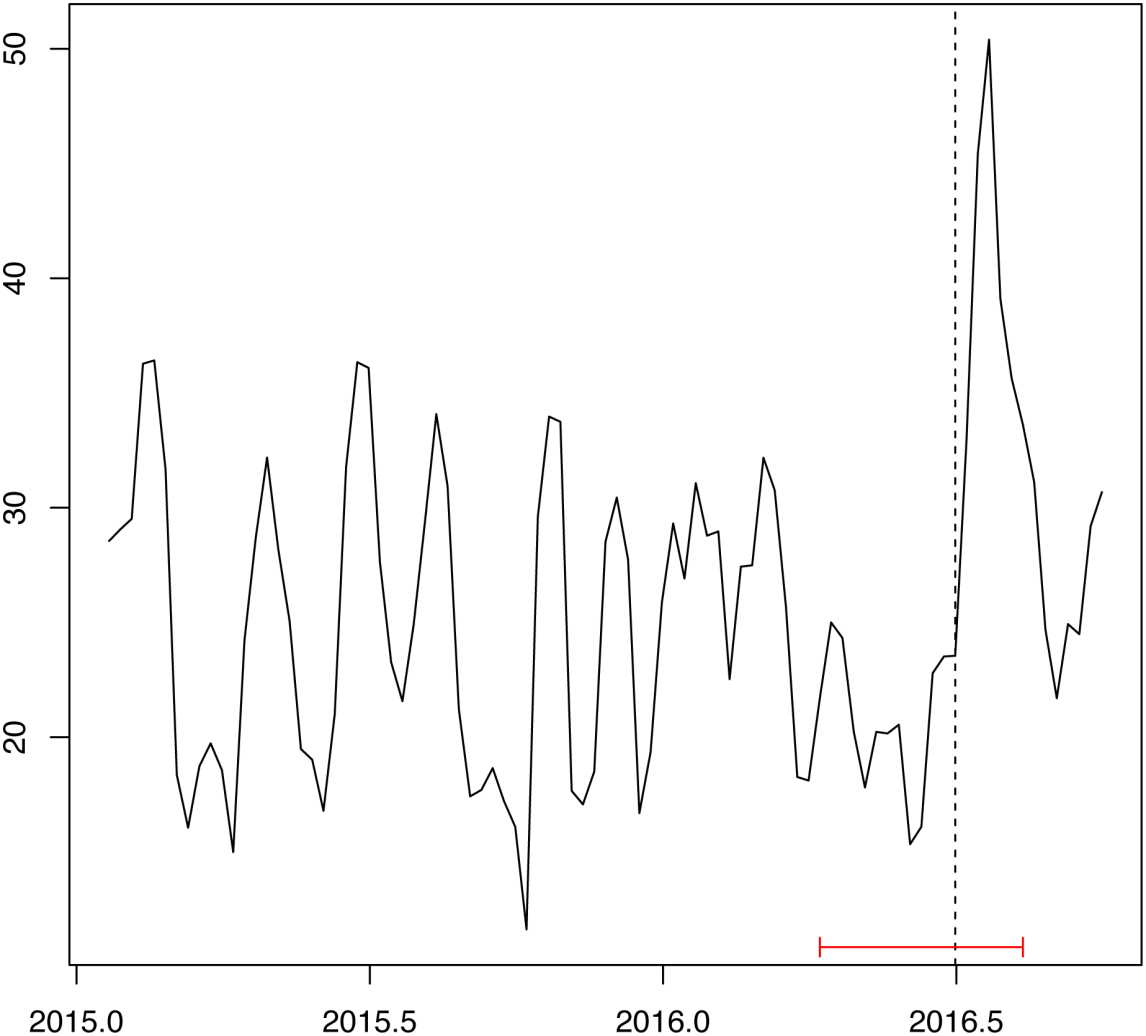
Twitter historical volatility.

The next step in the estimation procedure is to identify regimes in which the relative variances of the structural shocks have changed over time. To do so, we study the behavior of historical volatilities since they are expected to reflect such changes. In this case, the weekly historical volatility was calculated using daily data with 20-day windows. To systematically find a regime change, structural break tests are conducted in the historical volatility of Twitter data. We used the [39] test to find multiple breaks, allowing up to 5 breaks, and used a trimming of at least 0.15, so that each segment has a minimum of 15% of the observations in the sample. The best number of breaks was selected based on the Bayesian information criterion (BIC). Results are depicted in Fig 4.

The dotted line indicates the break date, and the horizontal red lines represent the 95% confidence bands for the identified break. The high volatility regime for the Twitter data occurs after July 1, 2016. Therefore, the period before this date is defined as the low volatility regime. To make sure we have correctly identified high and low volatility regimes, we can test whether the variances of *ε*_law_, *ε*_ctz_ and *ε*_twt_ in the high volatility regime are systematically larger than the corresponding variances in the low volatility regime. Results of this test are presented below.

Once the regimes have been identified, we proceed with the estimation of two different structural models. Model 1 includes law enforcement killed by citizens, number of minorities killed by police, and BLM tweets. Model 2 includes law enforcement killed by citizens, number of white non-Hispanics killed by police, and BLM tweets. By estimating these models we can test if there are differences in police reactions following a one-time structural shock to each group. Note that this procedure is carried out in four steps. In the first step, the reduced form VAR model is estimated as described in system (2). Based on the Akaike Information Criterion (AIC), the lag length of 2 weeks (*p* = 2) was selected. In the second step, model residuals are obtained and used to construct the variance-covariance matrix for both high and low volatility regimes. In the third step, these variance-covariance matrices are used to estimate system (6). Results from the estimation of this system are the contemporaneous coefficients and structural shock variances. The standard errors are calculated using a fixed-design wild bootstrap with 500 replications. Finally, after recovering structural parameters, we are able to conduct the analysis of impulse response functions.

Results from the estimation of contemporaneous parameters and structural shock variances are presented in Tables 4 and 5. Recall that the difference between model 1 and 2 is the racial/ethnic background of the citizens killed by police. Model 1 uses minorities, while model 2 uses white non-Hispanics. First, we focus on the ratios of the estimated variances of structural shocks from system (6). These are used to verify we achieved identification as the variances in the high volatility regime are expected to be greater than those in the low volatility regime. Therefore, *p*-values for the tests of the null hypotheses $H_{0}\text{:}\;\text{var}\left(\varepsilon_{g}^{\left(1\right)}\right)/\text{var}\left(\varepsilon_{g}^{\left(2\right)}\right)\leq 1$ for *g* = law, ctz, twt, are included. The bootstrapped *p*-values are less than 0.01 in all cases, except for *minorities* in model 1. This result is expected because the high volatility regime for this variable was regime 2. In both models, the variance of structural shocks for the Twitter data is three times larger in regime 1 (high volatility) compared to regime 2 (low volatility). This is to be expected since public opinion post July 1, 2016 is captured by an increased variation of BLM tweet changes.

**Table 4:** Model 1 (law, minorities and twitter).

	*Coefficient*	*p*–*value*^(a)^
*δ*_12_(minorities → law)	-0.0928	0.0000
*δ*_13_(twitter → law)	1.2525	0.0000
*δ*_21_ (law → minorities)	0.6280	0.0000
*δ*_23_ (twitter → minorities)	0.8174	0.0000
*δ*_31_ (law → twitter)	-0.2744	0.0000
*δ*_32_ (minorities → twitter)	-0.0005	0.9082
$\text{var}(\varepsilon_{law}^{\left(1\right)})$	0.0970	0.0000
$\text{var}(\varepsilon_{minorities}^{\left(1\right)})$	0.3303	0.0000
$\text{var}(\varepsilon_{twitter}^{\left(1\right)})$	0.0415	0.0000
$\text{var}(\varepsilon_{law}^{\left(2\right)})$	0.0582	0.0000
$\text{var}(\varepsilon_{minorities}^{\left(2\right)})$	0.4317	0.0000
$\text{var}(\varepsilon_{twitter}^{\left(2\right)})$	0.0118	0.0000
		*p*–*value*^(b)^
$\text{var}\left(\varepsilon_{law}^{\left(1\right)}\right)/\text{var}\left(\varepsilon_{law}^{\left(2\right)}\right)$	1.6653	0.0000
$\text{var}\left(\varepsilon_{minorities}^{\left(1\right)}\right)/\text{var}\left(\varepsilon_{minorities}^{\left(2\right)}\right)$	0.8014	1.0000
$\text{var}\left(\varepsilon_{twitter}^{\left(1\right)}\right)/\text{var}\left(\varepsilon_{twitter}^{\left(2\right)}\right)$	3.2140	0.0000

(*a*) *p*-value corresponds to the test of the null hypothesis *H*_0_:*δ*_*jk*_ = 0 ∀ *j* = 1, 2, 3 and *k* = 1, 2, 3.

(*b*) *p*value corresponds to test of the null hypothesis $H_{0}\text{:}\;\text{var}\left(\varepsilon_{g}^{\left(1\right)}\right)/\text{var}\left(\varepsilon_{g}^{\left(2\right)}\right)\leq 1$, for *g* = law, minorities, twitter

**Table 5:** Model 2 (law, whites and twitter).

	*Coefficient*	*p*–*value*^(a)^
*δ*_12_ (whites → law)	0.2015	0.0000
*δ*_13_ (twitter → law)	1.2116	0.0000
*δ*_21_ (law → whites)	-0.8281	0.0000
*δ*_23_ (twitter → whites)	-0.0181	0.8367
*δ*_31_ (law → twitter)	-0.2641	0.0000
*δ*_32_ (whites → twitter)	-0.0370	0.0000
$\text{var}(\varepsilon_{law}^{\left(1\right)})$	0.0925	0.0000
$\text{var}(\varepsilon_{whites}^{\left(1\right)})$	0.2672	0.0000
$\text{var}(\varepsilon_{twitter}^{\left(1\right)})$	0.0423	0.0000
$\text{var}(\varepsilon_{law}^{\left(2\right)})$	0.0531	0.0000
$\text{var}(\varepsilon_{whites}^{\left(2\right)})$	0.2547	0.0000
$\text{var}(\varepsilon_{twitter}^{\left(2\right)})$	0.0121	0.0000
		*p*–*value*^(b)^
$\text{var}\left(\varepsilon_{law}^{\left(1\right)}\right)/\text{var}\left(\varepsilon_{law}^{\left(2\right)}\right)$	1.7566	0.0000
$\text{var}\left(\varepsilon_{whites}^{\left(1\right)}\right)/\text{var}\left(\varepsilon_{whites}^{\left(2\right)}\right)$	1.0593	0.022
$\text{var}\left(\varepsilon_{twitter}^{\left(1\right)}\right)/\text{var}\left(\varepsilon_{twitter}^{\left(2\right)}\right)$	3.1639	0.0000

(*a*) *p*-value corresponds to the test of the null hypothesis *H*_0_:*δ*_*jk*_ = 0 ∀ *j* = 1, 2, 3 and *k* = 1, 2, 3.

(*b*) *p*value corresponds to test of the null hypothesis $H_{0}\text{:}\;\text{var}\left(\varepsilon_{g}^{\left(1\right)}\right)/\text{var}\left(\varepsilon_{g}^{\left(2\right)}\right)\leq 1$, for *g* = law, minorities, twitter

In model 1, the variance of law enforcement killed is 66% higher in regime 1 than in regime 2 (0.0970 vs. 0.0582). This means that post July 1, 2016 events are associated with a higher variation in the number of officers killed. Given that this series (law enforcement killed) is stationary with a zero lower bound, an increase in the variation can only come from the increased killings of police officers. This claim is supported by the Dallas shootings in July 7, 2016. In the same model, the variance of minorities killed by police is 23% smaller in regime 1 compared to regime 2 (0.3303 vs. 0.4317). This indicates that the events post July 1, 2016 are associated with a decrease in the number of minorities killed by police. Again, this series is stationary with a zero lower bound, therefore a decrease in variance can only come from the decrease in the number of minorities killed. This indicates that the low volatility regime for minorities shot and killed by law enforcement corresponds to the high volatility regime for Twitter and police officers shot and killed by citizens.

In model 2, the variance of law enforcement killed is 74% higher in regime 1 than in regime 2. This is consistent with model 1. In the same model, the variance of white non-Hispanic citizens killed by law enforcement is 5% higher in regime 1 than in regime 2 (0.2672 vs. 0.2547). Even though the ratio is statistically significant, it is small from a practical point of view. This tells us that the relative frequency of law enforcement officers shooting white non-Hispanics did not substantially change in the post July 1, 2016 time frame even though the number of minorities killed in the same period decreased, on average. Overall, these results indicate that the large increase in the variances of the structural shocks in the selected high volatility regime, specifically the BLM twitter activity, is sufficient to achieve identification. Following Lanne, Lütkepohl and Maciejowska [40] we also tested for the uniqueness requirement to check if identification has been achieved. It implies that the change in the relative variance should not be homogeneous across variables in the system. The null hypothesis tested is:

$$\begin{array}{c} H_{0}\text{:}\;\frac{\text{var}(\varepsilon_{j}^{\left(1\right)})}{\text{var}(\varepsilon_{k}^{\left(1\right)})}/\frac{\text{var}(\varepsilon_{j}^{\left(2\right)})}{\text{var}(\varepsilon_{k}^{\left(2\right)})}\neq 1\;\text{for}\;j\neq k\in \left[\text{law,}\;\text{ctz,}\;\text{twt}\right] \end{array}$$

and was conducted using the bootstrap results. The results confirmed that we have achieved identification.

Continuing with Table 4, we now focus on the estimates of contemporaneous coefficients (off-diagonal elements of matrix *A*^−1^). The *p*-values for the tests of statistical significance of contemporaneous effects *H*_0_: *δ*_*jk*_ = 0 for *j* = 1, 2, 3 and *k* = 1, 2, 3 such that *j* ≠ *k* are also reported. All parameters, with exception of the effect of a shock to minorities killed on the number of BLM tweets, are statistically significant at the 0.01 level.

- On average, a 1% increase in the number of minorities killed (MINORITIES) is associated with a 0.09% decrease in the number of law enforcement deaths (LAW) the same day the shock occurs. The same shock has no effect on the growth rate of BLM tweets (TWITTER).
- A 1% shock (increase) to LAW increases MINORITIES by 0.63%, and decreases the growth rate of TWITTER by 27% the same day the shock occurs, on average.
- On average, a 1% shock (increase) to TWITTER increases LAW by 1.25%, and increases MINORITIES by 0.82% the same day the shock occurs.

Focusing on the contemporaneous coefficients in model 2 (Table 5), we can observe that all of the parameters, with the exception of the effect of a shock to the BLM tweets on the number of white non-Hispanics killed by law enforcement, are statistically significant at 0.01 level. One can observe that some shocks have different effects either in magnitude or sign than in model 1.

- On average, a 1% increase in the number of white non-Hispanics (WHITES) is associated with a 0.2% increase in LAW on the same day. Compare this to model 1 where the same 1% shock to MINORITIES reduces the number of dead police officers by 0.09%.
- A 1% shock to LAW decreases WHITES on the same day by 0.83%, on average. This is different from model 1 where the same shock increases MINORITIES by 0.63%.
- A 1% shock to TWITTER has no effect on WHITES on the same day (coefficient is statistically insignificant), yet the same shock increases the MINORITIES by 0.82% in model 1.
- A 1% shock to WHITES causes a 0.04% drop in TWITTER, yet the same 1% shock to MINORITIES killed has no statistically significant effect on TWITTER in model 1.

## Impulse response analysis

**Fig 5:**
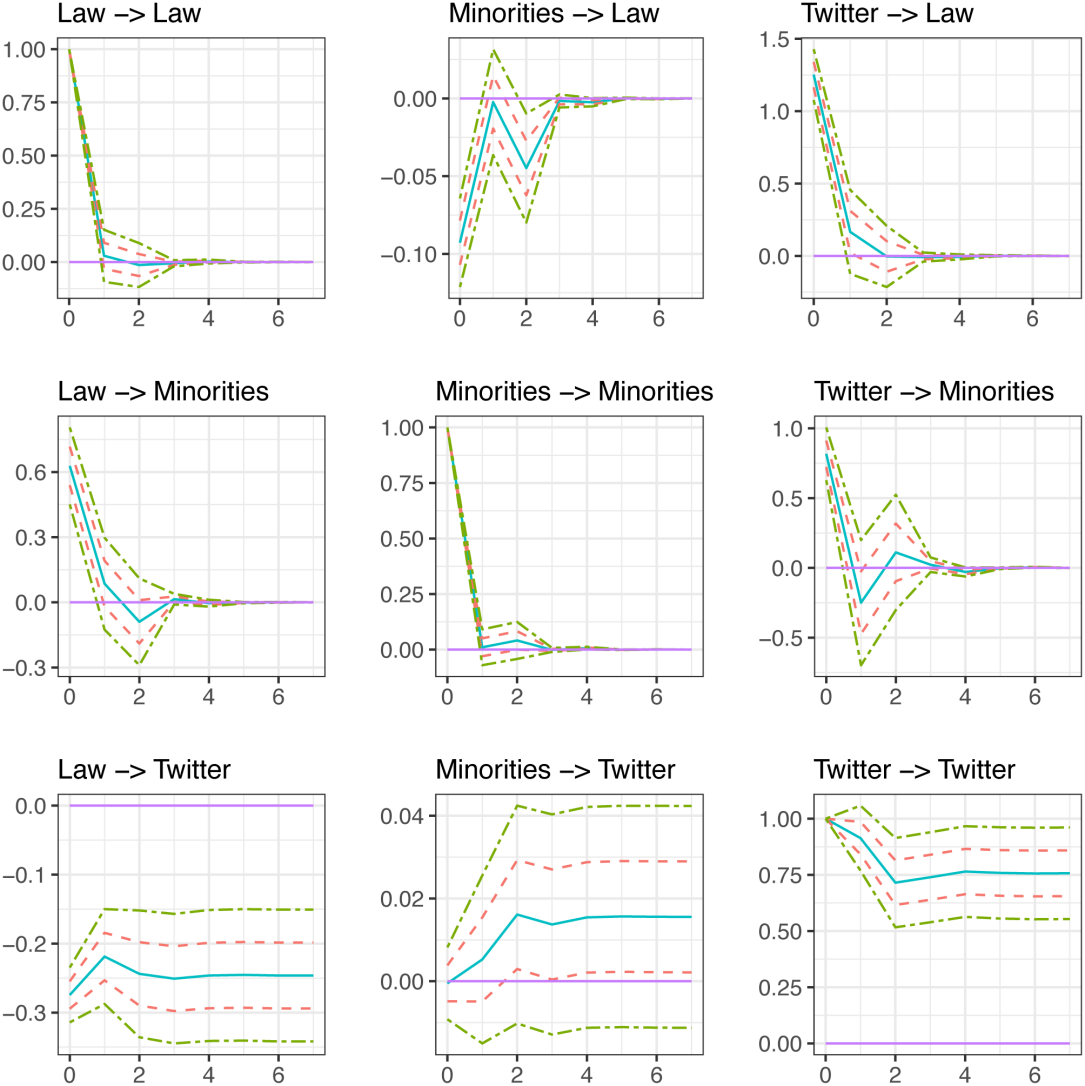
Impulse response functions (model 1).

**Fig 6:**
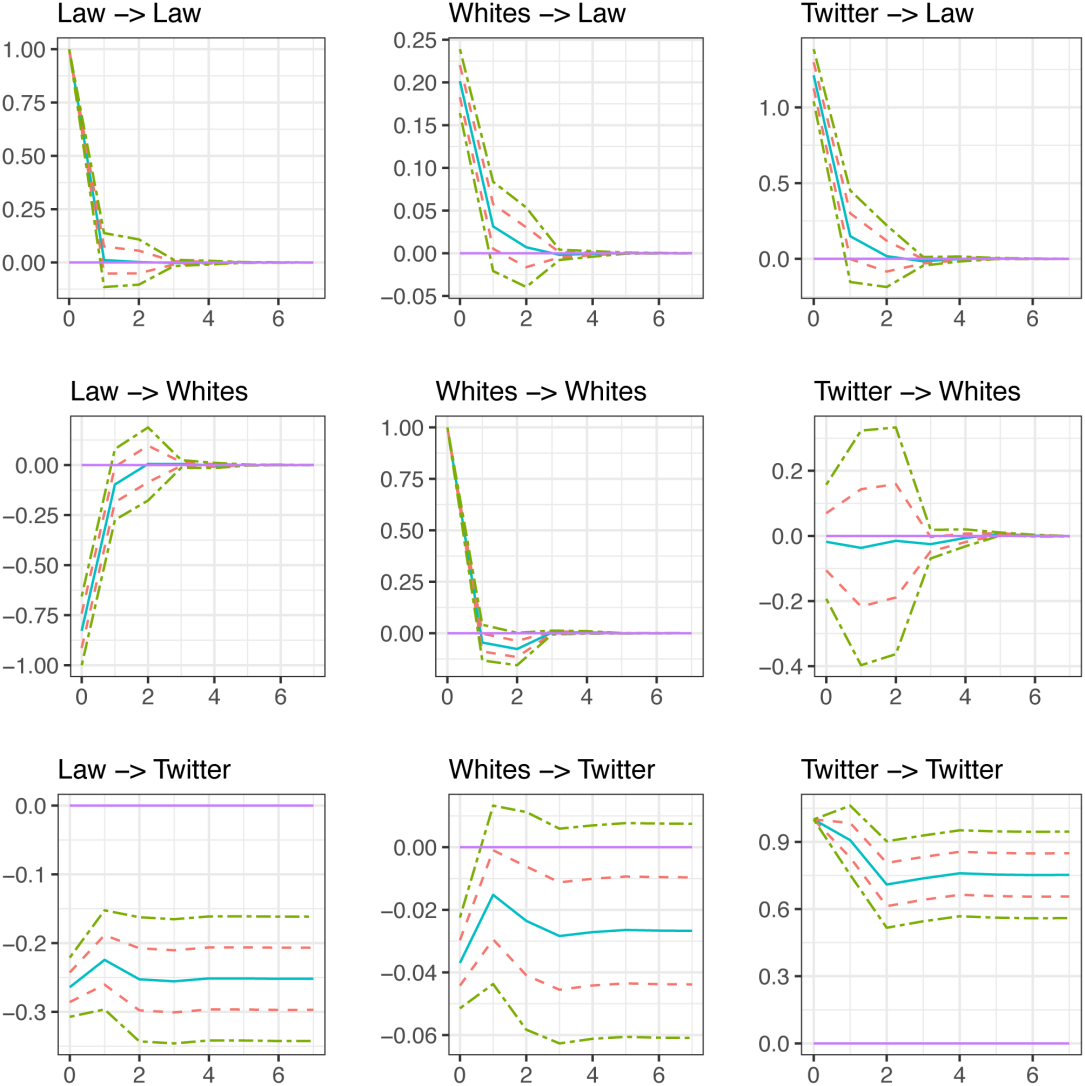
Impulse response functions (model 2).

After identifying the contemporaneous effects in each system, we are now interested in evaluating the total effect, contemporaneous and lagged, of a 1% shock to each of the endogenous variables on itself and on the rest of the endogenous variables in the system. To do this, we focus on the calculation of impulse response functions (IRF). Figs 5 and 6 present the IRFs that were calculated based on the identification through heteroskedasticity scheme described earlier. This allows us to run through a sequence of scenarios to examine, for example, what would happen to the number of minorities killed the day of, or any reasonable arbitrary number of days after, an unexpected increase in the number of law enforcement officers killed by citizens. Or we can ask, what effect a shock to BLM tweets will have on the number of law enforcement killed by citizens and the number of citizens killed by law enforcement? We plot the IRF based on the original estimation from the data (solid line) as opposed to the median or mean IRF from the simulated data (in both cases the results are very similar), as well as one and two standard deviation bands represented by dashed and dot-dashed lines, respectively. The X-axis represents the number of days from the initial shock to the variable of interest. Here, we plot impulse responses up to 7 days after the shock. An effect is considered statistically significant for the period of time in which the upper and lower 95% confidence bands are either above or below zero. If the 95% confidence interval includes zero, the response of the variable in question is statistically insignificant, meaning that the response of that variable to a particular structural shock is null. Since the TWITTER variable is in first differences, while the rest of the variables are in levels, we plot the cumulative IRF of this variable to a 1% shock to each of the endogenous variables in the model to be consistent with the interpretation of the IRF of the variables that are in levels.

### Fatal law enforcement & minority civilian encounters

The first relationship of significance from Fig 5 includes LAW, which has an immediate and significant positive association with MINORITIES that becomes insignificant after the first day. This means that an unexpected 1% shock to the number of law enforcement officers shot to death in a day is associated with a 0.63% increase in the number of minority citizens shot to death on the same day, on average. Alternatively, a 100% increase in LAW is associated with a 63% increase in MINORITIES, net any effect attributed to the number of tweets that reference Black Lives Matter. In practical terms, if the number of law enforcement officers killed in one day doubles from the average, there are an additional 0.8 minorities shot to death for one day.

MINORITIES has an immediate and significant negative association with LAW that becomes insignificant after the first day, which is associated with a 0.09% decrease in the number of law enforcement officers shot to death. In practical terms, if the number of minorities killed in one day doubles from the average, there are in 0.01 fewer law enforcement officers shot to death for one day. TWITTER has an immediate and significant positive association with MINORITIES that becomes insignificant after the first day, which is associated with an 0.82% increase in the number of minority citizens shot to death on the same day. In practical terms, if the number of tweets related to Black Lives Matter doubles from the average, there are an additional 1.02 minorities shot to death that day. Similarly, TWITTER has an immediate and significant positive association with LAW that becomes insignificant after the first day, which is associated with an increase in the number of law enforcement shot to death in a day by more than 1.25%. In practical terms, if the number of tweets related to Black Lives Matter doubles from the average, there are an additional 0.16 law enforcement officers shot to death that day.

### Fatal law enforcement & white-non Hispanic civilian encounters

Fig 6 mirrors the model presented in the previous subsection, however, the number of minorities shot to death by law enforcement is replaced with the number of white non-Hispanics shot to death by law enforcement (WHITE). LAW has an immediate and significant negative association with WHITE that becomes insignificant after the first day. An unexpected 1% shock to the number of law enforcement officers shot to death in a day is associated with a 0.83% decrease in the number of white non-Hispanic citizens shot to death on the same day. Or, a 100% increase in LAW is associated with an 83% decrease in WHITE, net any effect attributed to the number of tweets that reference Black Lives Matter. In practical terms, if the number of law enforcement officers shot to death doubles from the average, there are 1.2 fewer white non-Hispanics shot to death that day.

WHITE has an immediate and significant positive association with LAW that becomes insignificant after the first day, which is associated with a 0.2% increase in the number of law enforcement officers shot to death on the same day. In practical terms, if the number of white non-Hispanics shot to death doubles from the average, there are an additional 0.03 law enforcement officers shot to death that day. Moreover, TWITTER has an immediate and significant positive impact on LAW that becomes insignificant after the first day, which is associated with an increase in the number of law enforcement shot to death in a day by more than 1.2%. In practical terms, if the number of tweets related to Black Lives Matter doubles from the average, there are an additional 0.16 law enforcement officers shot to death that day.

Also of interest, was the relationship between these shooting deaths and both the social media and general media coverage of law enforcement/citizen fatal encounters. An unexpected shock to law enforcement killed is associated with a significant, immediate and prolonged decrease in the amount of media content published in both models. This same media suppression pattern appeared when white non-Hispanics was shocked. A shock to the number of minorities shot to death had no relationship with the number of tweets referencing Black Lives Matter. These results demonstrate that law enforcement and white deaths are associated with less coverage within all types of media, while minority deaths had no relationship.

## Results & implications

Using a structural vector-autoregression framework, we analyzed daily data between January 1, 2015 and September 30, 2016, modeling the contemporaneous, cyclical relationship between the number of law enforcement officers shot to death in the United States, the number of citizens shot and killed by law enforcement in the United States, and the number of tweets that included #BlackLivesMatter or the term “Black Lives Matter.” Two models were run, separating citizens killed into minorities and white non-Hispanics (See the Supporting information section for additional model information). Our results provide evidence of a retaliatory, violent relationship between law enforcement and citizens. Unexpected shocks to the number of law enforcement officers killed are associated with more minorities killed and fewer whites killed on the same day. In addition, our models found that unexpected increases in citizen deaths increased the number of law enforcement officers killed if the citizens were white non-Hispanics, and decreased the number of law enforcement officers killed if the citizens were minorities. These relationships held regardless of how much social media attention was focused on the Black Lives Matter movement.

In an attempt to understand our results, we present two possible explanations for these homicide patterns taken from the economics and psychology literature. The rational choice perspective (see [41, 42]) is the simplest and most direct explanation for the reduction in the number of officers shot and killed in the line of duty after a minority citizen is shot and killed. Based on the assumption that humans are rational beings who engage in cost-benefit analyses of their behaviors, rational choice theorists would argue that, when controlling for the amount of social media exposure, minority citizens would be cognizant of the high cost of an encounter with law enforcement officers after learning of the deaths of other members of the public who are minorities. In turn, this would alter their behaviors to reduce the likelihood of contact with law enforcement and, if contacted, increase behaviors that would reduce the risk of the encounter turning deadly. This would reduce the risk of routine contacts between law enforcement and the public escalating into a situation where an officer is killed in the line of duty.

For a possible explanation of the relationship between law enforcement killed and minorities killed, we turn to the field of psychology and terror management theory, which posits that when individuals are death primed—that is, their mortality is either consciously or subconsciously salient—they are more likely to support the predominant cultural worldview of the society in which they live and react positively to those associated with ingroups of that worldview, and negatively to those who they associate as being part of outgroups [43]. This worldview defense has also been shown to increase levels of punitiveness [44]. Mortality salience does not have an equal impact on all individuals, but its effects have been shown to be more pronounced on those with lower self-esteem [45]. Experimental research has also shown that ingroup/outgroup identification extends to race as, in one study, whites were more likely to view white racists more positively when mortality was salient [46].

Law enforcement officers are hired and trained to support the dominant cultural worldview of the communities they serve through the enforcement of codified legal norms. Any increase in mortality salience should increase their defense of that worldview. In addition, the Black Lives Matter movement (and minorities through association) could be viewed as an outgroup, threatening the predominant worldview and the established culture of the criminal justice system. Terror management theory would assert that if law enforcement officers are made aware of the homicides of their colleagues, this death prime would increase their mortality salience and alter the ways in which they engage with individuals from perceived outgroups in order to defend their cultural worldview. This effect would only be temporary and would explain why the increase in the number of minorities killed is only significant on the same day in our models. In addition, officers who are death primed may underestimate the risk posed by those from their perceived ingroup (such as whites) and be less prepared to use fatal force against these individuals if a contact requires. This would explain the significant decrease in the number of white non-Hispanics killed after an officer is killed in the line of duty.

We also found evidence of social media incitement. In both models, an unexpected shock to the number of tweets referencing the Black Lives Matter movement is associated with a significant and immediate increase in the number of law enforcement officers shot to death. The results measuring the relationship between social media and shootings of citizens were not as robust as an unexpected shock to the number of tweets referencing Black Lives Matter, which was associated with an immediate increase in the number of minorities killed by law enforcement, but had no relationship with the number of white non-Hispanics killed.

## Conclusion

This study set out to examine whether there was evidence of retaliatory fatal violence between law enforcement officers and the public, while controlling for the prevalence of social media connected to the Black Lives Matter movement. The results show that there does appear to be retaliatory violence; specifically, that an unexpected shock to the number of law enforcement officers shot in the line of duty is associated with a significant increase in the number of minorities killed in the same day. However, unexpected shocks to minorities shot by law enforcement actually are associated with a significant decrease in the number of law enforcement officers shot, while shocks to white-non Hispanics shot by law enforcement are associated with increases in law enforcement officers killed. We frame these results through both terror management theory and rational choice theory although, to be clear, we did not explicitly operationalize and test these theories. Also, we acknowledge that our theoretical explanations are not definitive and competing explanations are plausible. For example, as one reviewer noted, police violence against citizens could be a sign of civil unrest, which law enforcement officers may interpret as an increase in risk to themselves and their colleagues, leading to a heightened use of defensive tactics that explains why no pattern of retaliatory violence was identified against law enforcement when a minority member of the public was shot.

Specific to social media as a contagion, we found that an unexpected shock to tweets related to Black Lives Matter are associated with increases in both the numbers of law enforcement officers and minorities shot and killed. These results can be framed through research demonstrating that social media can spread negative affect and can do so quickly depending on the nature of the communication’s message.

While we would hesitate to draw firm policy implications until replication of the core findings as well as testing of policy options can be undertaken, our results suggest some potential directions that might be investigated in future research. First, although we do not claim that the observed increase in law enforcement shootings of minorities are non-justifiable uses of deadly force, this relationship between minorities and law enforcement signals the possibility of a disproportionate reaction to public killings of law enforcement officers based on race, especially when compared to the observed contrary relationship between whites and law enforcement. Future research might consider these results and how to address them in the context of:

- Training: for example, experimentally manipulating mortality salience in order to explore its effect on trainee performance, and exploring training curricula that make officers aware of these patterns and provide guidance on how to mitigate the effects of social media contagion
- Policies: for example, testing whether an agency’s current systematic communications about officers killed in the line of duty across the country (which might unintentionally and needlessly increase the mortality salience of officers) could be limited, and with what effect, unless a specific threat to their agency and personnel exists
- Levels of community engagement: for example, experimenting with providing officers opportunities to engage in meaningful outreach with minority communities to limit officer perceptions of minorities as being part of an outgroup.

Second, although the First Amendment to the U.S. Constitution guarantees all Americans the freedom of speech, those who employ social media to advocate on behalf of, or against, social movements should also be cognizant of the real world impact of their communiques. While we would not suggest any undue restrictions on free speech, we note that similar to the oft-invoked example of yelling “Fire!” in a crowded theater where no fire exists, there are common sense limits to free speech that may apply to ill-considered social media declarations of a “war on cops” or of “racist” police. Those who have used the Black Lives Matter hashtag or terminology in ways meant to either empower or disenfranchise the movement may have brought along with their messages a heightened awareness of the life endangering conflict that occurs between law enforcement officers and minority communities and, in doing so, appear to increase the risk to both.

## Supporting information

S1 Appendix Multivariate dynamic analysis. Structural VAR Models and the Identification Problem.
(PDF)

S1 Data File Data used for the analysis. (CSV)
