# Supplementary material for: Primed for death: Law enforcement-citizen homicides, social media, and retaliatory violence
Source: PLoS One. 2018 Jan 10;13(1):e0190571. doi: 10.1371/journal.pone.0190571 (PMC5761867; doi:10.1371/journal.pone.0190571)
Supplement: S1 Appendix — Structural VAR Models and the Identification Problem. (PDF) [file pone.0190571.s001.pdf]

Supporting Information for "Primed for Death: Law Enforcement-Citizen Homicides, Social Media, and Retaliatory Violence"

Vladimir Bejan[1], Matthew Hickman[2], William S. Parkin[2,*], Veronica F. Pozo[3]

1 Department of Economics, Seattle University, Seattle, WA 98122 (USA)

2 Department of Criminal Justice, Seattle University, Seattle, WA 98122 (USA)

3 Department of Applied Economics, Utah State University, Logan, UT 84322 (USA)

* Corresponding author. Email: parkinw@seattleu.edu

S1 Appendix: Multivariate dynamic analysis

Structural VAR Models and the Identification Problem

SVAR models were first proposed by Sims [1] as an alternative to traditional large-scale dynamic simultaneous equations models. Structural inference in VAR models requires differencing between correlation and causation. The generation of such interpretation is known as identification of a model [2]. To illustrate the identification problem, consider the following bivariate one lag system:

$$\begin{aligned} Y_t &= a_0 + a_1 X_t + a_2 Y_{t-1} + a_3 X_{t-1} + \varepsilon_{t,Y} \\ X_t &= b_0 + b_1 Y_t + b_2 Y_{t-1} + b_3 X_{t-1} + \varepsilon_{t,X} \end{aligned} \tag{1}$$

where both endogenous variables Y_t and X_t have contemporaneous effects on each other, and $\varepsilon_{t,Y}$ and $\varepsilon_{t,X}$ are uncorrelated white noise shocks with standard deviations equal to 1. Equation (1) cannot be consistently estimated in its current form because it incorporates feedback (i.e., model variables are allowed to affect each other). For simplicity, we re-write equation (1) in a matrix form:

$$\begin{bmatrix} 1 & -a_1 \\ -b_1 & 1 \end{bmatrix} \begin{bmatrix} Y_t \\ X_t \end{bmatrix} = \begin{bmatrix} a_0 \\ b_0 \end{bmatrix} + \begin{bmatrix} a_2 & a_3 \\ b_2 & b_3 \end{bmatrix} \begin{bmatrix} Y_{t-1} \\ X_{t-1} \end{bmatrix} + \begin{bmatrix} \varepsilon_{t,Y} \\ \varepsilon_{t,X} \end{bmatrix}$$

which is equivalent to:

$$AZ_t = \Gamma_0 + \Gamma_1 Z_{t-1} + \varepsilon_t$$

where $A = \begin{bmatrix} 1 & -a_1 \\ -b_1 & 1 \end{bmatrix}$, $Z_t = \begin{bmatrix} Y_t \\ X_t \end{bmatrix}$, $\Gamma_0 = \begin{bmatrix} a_0 \\ b_0 \end{bmatrix}$, $\Gamma_1 = \begin{bmatrix} a_2 & a_3 \\ b_2 & b_3 \end{bmatrix}$ and $\varepsilon_t = \begin{bmatrix} \varepsilon_{t,Y} \\ \varepsilon_{t,X} \end{bmatrix}$. The reduced form VAR is obtained by pre-multiplying equation () by A^{-1}

$$Z_t = A^{-1}\Gamma_0 + A^{-1}\Gamma_1 Z_{t-1} + A^{-1}\varepsilon_t$$

This model can be re-written as:

$$Z_t = D_0 + D_1 Z_{t-1} + e_t \tag{2}$$

where $D_0 = A^{-1}\Gamma_0$, $D_1 = A^{-1}\Gamma_1$ and $e_t = A^{-1}\varepsilon_t$. Equation (2) contains only lagged variables of the dependent variables. Here, the system does not contain contemporaneous effects and can be consistently estimated. Notice that reduced form errors e_t are a composite of the underlying structural shocks $\varepsilon_{t,Y}$ and $\varepsilon_{t,X}$.

$$e_t = A^{-1}\varepsilon_t$$

which is equivalent to:

$$\begin{bmatrix} e_{t,Y} \\ e_{t,X} \end{bmatrix} = \frac{1}{1 - a_1 b_1} \begin{bmatrix} 1 & a_1 \\ b_1 & 1 \end{bmatrix} \begin{bmatrix} \varepsilon_{t,Y} \\ \varepsilon_{t,X} \end{bmatrix} \tag{3}$$

Similarly, it can be shown that the estimated reduced form parameters D_0 and D_1 are functions of the underlying structural parameters A, Γ_0 and Γ_1. Thus, one cannot interpret reduced form coefficients as if they were structural ones. To examine the structural relationship between variables, which is the main goal of our analysis, it is

necessary to recover structural parameters using reduced form estimates. That is, we first need to recover the elements of A^{-1}. This can only be achieved by imposing additional identification assumptions or restrictions.

To illustrate the identification problem, we start by understanding the relationship between the variances of e_t and ε_t. The variance of e_t is given by $\Sigma_e = E\left(e_t e_t^T\right)$, while the variance of ε_t is $\Sigma_\varepsilon = E\left(\varepsilon_t \varepsilon_t^T\right) = I$, by assumption. This relationship implies:

$$\Sigma_e = E\left(e_t e_t^T\right) = E\left(A^{-1}\varepsilon_t \left(A^{-1}\varepsilon_t\right)^T\right) = E\left(A^{-1}\Sigma_\varepsilon \left(A^{-1}\right)^T\right) = A^{-1}\left(A^{-1}\right)^T \qquad (4)$$

where the superscript T stands for the transpose of the corresponding matrix. Here, the reduced form covariance matrix has $K\left(K+1\right)/2$ unique elements since it is symmetric, while A^{-1} has potentially K^2 unique elements[1], where K is the number of endogenous variables in the VAR model. As a result, it is impossible to recover the elements of A^{-1} without imposing additional restrictions. Imposing $K\left(K-1\right)/2$ restrictions on the A^{-1} matrix allows us to recover the remaining elements using estimates of the reduced-form parameters. Note that the A^{-1} matrix is the contemporaneous effects matrix that shows how endogenous variables affect each other at time t. Imposing exclusion restriction implies that one variable does not affect another variable contemporaneously, but it does affect it with a lag. This is different from single equation time series models where a declaration of a dependent variable implies that it cannot affect explanatory variables even with a lag.

The most commonly used identification approach is Choleski decomposition which decomposes any positive definite $(m \times m)$ matrix into a product of a lower triangular matrix P with positive main diagonal and its transpose [3]. In our case, Choleski decomposition is equivalent to setting matrix A^{-1} to be lower triangular. Such identification imposes contemporaneous causality between the system endogenous variables. A shock to Y_t affects X_t contemporaneously, while a shock to X_t affects Y_t only with a lag. As a result, the behavior of impulse response functions will depend on the ordering of the variables, which is one of the main criticism of using this approach to identify structural VAR models. To solve the identification problem in this study, we

[1] We can normalize the diagonal elements of A^{-1} to one and this will leave us with $\left(K^2 - K\right)$ unique elements to estimate.

implement the method of identification through heteroskedasticity proposed by Rigobon [4].

Identification through heteroskedasticity

This method, based on the heteroskedasticity of the structural shocks, measures the contemporaneous relationship between variables by recognizing two regimes, one of high volatility and other of low volatility. Under a simple assumption of homoscedasticity, the system that represents the variance-covariance matrix of the reduced form residuals derived from equation (2) contains more unknowns than equations. The recognition of two regimes allows us to specify a system that has the same number of equations and unknowns (just identified system), which can be estimated by the generalized method of moments (GMM). There are two assumptions that lead to the identification of system (2): (i) parameters in structural equations are stable across the heteroskedasticity regimes (the variances are the ones changing), and (ii) structural shocks are not correlated [4]. The first one is the usual assumption imposed on ARCH or GARCH type models, and the second assumption is standard in the literature [5].

To illustrate the implementation of this identification approach, we focus on the relationship between the reduced form and structural shocks (equation 3). For simplicity, the A^{-1} matrix is defined as[2]:

$$A^{-1} = \begin{bmatrix} 1 & \delta_1 \\ \delta_2 & 1 \end{bmatrix}$$

Then, the reduced form residuals can be specified as:

$$e_{t,Y} = \varepsilon_{t,Y} + \delta_1 \varepsilon_{t,X}$$
$$e_{t,X} = \delta_2 \varepsilon_{t,Y} + \varepsilon_{t,X}$$

which shows how reduced form residuals are correlated. Therefore, a one-time shock to any of the variables included in a reduced form model will not tell us anything about the structure of their underlying system. To reconstruct $\varepsilon_{t,Y}$ and $\varepsilon_{t,X}$ it is necessary to

[2] It does not make a difference if we divide each element of the matrix by the determinant of A^{-1}, because it is a constant. Once the off diagonal elements of A^{-1} are identified, it is trivial to compute its determinant.

recover the elements of A^{-1} from consistent estimates of the reduced-form parameters. Under the assumptions of homoskedasticity of the structural shocks, we have:

$$\begin{cases} \mathrm{var}\left(e_{t,Y}\right) = \mathrm{var}\left(\varepsilon_{t,Y}\right) + \delta_1^2 \mathrm{var}\left(\varepsilon_{t,X}\right) \\ \mathrm{cov}\left(e_{t,Y}, e_{t,X}\right) = \delta_2 \mathrm{var}\left(\varepsilon_{t,Y}\right) + \delta_1 \mathrm{var}\left(\varepsilon_{t,X}\right) \\ \mathrm{var}\left(e_{t,X}\right) = \delta_2^2 \mathrm{var}\left(\varepsilon_{t,Y}\right) + \mathrm{var}\left(\varepsilon_{t,X}\right) \end{cases}$$

This is a system of three equations and four unknowns, so it is not possible to estimate it without additional restrictions. [4] built on the logic that by recognizing two or more regimes in the variances of the structural shocks, it is possible to identify the system with no further restrictions. Letting the superscript $i = 1, 2$ denote the regime, the following moment conditions can be specified:

$$\begin{cases} \mathrm{var}\left(e_{t,Y}^{(i)}\right) = \mathrm{var}\left(\varepsilon_{t,Y}^{(i)}\right) + \delta_1^2 \mathrm{var}\left(\varepsilon_{t,X}^{(i)}\right) \\ \mathrm{cov}\left(e_{t,Y}^{(i)}, e_{t,X}^{(i)}\right) = \delta_2 \mathrm{var}\left(\varepsilon_{t,Y}^{(i)}\right) + \delta_1 \mathrm{var}\left(\varepsilon_{t,X}^{(i)}\right) \\ \mathrm{var}\left(e_{t,X}^{(i)}\right) = \delta_2^2 \mathrm{var}\left(\varepsilon_{t,Y}^{(i)}\right) + \mathrm{var}\left(\varepsilon_{t,X}^{(i)}\right) \end{cases} \tag{5}$$

Here, there are six equations (three equations for each regime), and six unknowns: δ_1, δ_2, var $\left(e_{t,Y}^{(1)}\right)$, var $\left(e_{t,Y}^{(2)}\right)$, var $\left(e_{t,X}^{(1)}\right)$, and var $\left(e_{t,X}^{(2)}\right)$, indicating that the identification problem can be solved, with the assumption that δ_1 and δ_2 are stable across regimes. The variance-covariance matrix of the reduced form residuals can be computed for both regimes after estimating the reduced form Eq (2) by ordinary least squares (OLS). The coefficients in A^{-1} can be estimated by GMM using system (5) as moment conditions, and standard errors can be obtained using the fixed-design wild bootstrap [6] with the Rademacher distribution as the pick distribution [7].

References

1. Sims CA. Macroeconomics and reality. Econometrica: Journal of the Econometric Society. 1980; p. 1–48.

2. Sims CA. Are forecasting models usable for policy analysis? Federal Reserve Bank of Minneapolis Quarterly Review. 1986;10(1):2–16.

3. Lütkepohl H. New introduction to multiple time series analysis. Springer Science & Business Media; 2005.

4. Rigobon R. Identification through heteroskedasticity. Review of Economics and Statistics. 2003;85(4):777–792.

5. Ehrmann M, Fratzscher M, Rigobon R. Stocks, bonds, money markets and exchange rates: measuring international financial transmission. Journal of Applied Econometrics. 2011;26(6):948–974.

6. Goncalves S, Kilian L. Bootstrapping autoregressions with conditional heteroskedasticity of unknown form. Journal of Econometrics. 2004;123(1):89–120.

7. Godfrey L. Bootstrap tests for regression models. Palgrave Macmillan; 2009.
